# DHFR-mediated effects of methotrexate in medulloblastoma and osteosarcoma cells: The same outcome of treatment with different doses in sensitive cell lines

**DOI:** 10.3892/or.2015.3819

**Published:** 2015-02-26

**Authors:** JAKUB NERADIL, GABRIELA PAVLASOVA, MARTIN SRAMEK, MICHAL KYR, RENATA VESELSKA, JAROSLAV STERBA

**Affiliations:** 1 Department of Experimental Biology, School of Science, Masaryk University, Brno, Czech Republic; 2 Regional Centre for Applied Molecular Oncology, Masaryk Memorial Cancer Institute, Masaryk University, Brno, Czech Republic; 3 Department of Pediatric Oncology, University Hospital Brno and School of Medicine, Masaryk University, Brno, Czech Republic

**Keywords:** methotrexate, leucovorin, osteosarcoma, resistance, antifolate, medulloblastoma

## Abstract

Although methotrexate (MTX) is the most well-known antifolate included in many standard therapeutic regimens, substantial toxicity limits its wider use, particularly in pediatric oncology. Our study focused on a detailed analysis of MTX effects in cell lines derived from two types of pediatric solid tumors: medulloblastoma and osteosarcoma. The main aim of this study was to analyze the effects of treatment with MTX at concentrations comparable to MTX plasma levels in patients treated with high-dose or low-dose MTX. The results showed that treatment with MTX significantly decreased proliferation activity, inhibited the cell cycle at S-phase and induced apoptosis in Daoy and Saos-2 reference cell lines, which were found to be MTX-sensitive. Furthermore, no difference in these effects was observed following treatment with various doses of MTX ranging from 1 to 40 *μ*M. These findings suggest the possibility of achieving the same outcome with the application of low-dose MTX, an extremely important result, particularly for clinical practice. Another important aspect of treatment with high-dose MTX in clinical practice is the administration of leucovorin (LV) as an antidote to reduce MTX toxicity in normal cells. For this reason, the combined application of MTX and LV was also included in our experiments; however, this application of MTX together with LV did not elicit any detectable effect. The expression analysis of genes involved in the mechanisms of resistance to MTX was a final component of our study, and the results helped us to elucidate the mechanisms of the various responses to MTX among the cell lines included in our study.

## Introduction

Methotrexate (MTX) is the most widely known antifolate successfully used in oncology for a long time. MTX is particularly effective in the treatment of acute lymphoblastic leukemia, non-Hodgkin lymphoma, breast carcinoma, lung carcinoma, osteosarcoma, choriocarcinoma, and some neuroectodermal tumors (1). Although MTX has been included in therapeutic protocols for more than 60 years, its dosage as well as administration schedules are still being optimized.

The most important effect of MTX is based on the inhibition of dihydrofolate reductase (DHFR), which blocks the reduction of folic acid and, consequently, folic acid metabolism. When the concentration of MTX exceeds the binding capacity of DHFR, all available molecules of tetrahydrofolate (THF) are gradually depleted in the cell, and the synthesis of purine and pyrimidine precursors, which are necessary for synthesis of nucleic acids, is reduced (2).

Although MTX is included in many standard therapeutic regimens, its substantial toxicity limits its wider use, particularly in pediatric oncology. The cytotoxic effects of high-dose MTX (HD-MTX) on normal somatic cells could be reduced by the administration of an antidote, with the most frequent being leucovorin (LV). Another possibility of an MTX schedule is the repeated administration of low-dose MTX (LD-MTX) without LV (3,4).

Nevertheless, there is a fear in clinical practice that HD-MTX chemotherapy can induce drug resistance, resulting in a reduced treatment effect (5). The primary and the most frequent mechanism of resistance to MTX is caused by defects in reduced folate carrier (RCF)-mediated transport, which are caused by mutations in the *RCF* gene or by the downregulation of its expression (6). Other well-described mechanisms of MTX resistance include the overexpression of DHFR or thymidylate synthase (TYMS) or mutations in genes encoding these enzymes, decreasing their affinity for antifolates. Another important aspect in resistance to MTX is defective polyglutamylation, which substantially reduces the cytotoxicity of MTX. Reductions in MTX polyglutamylation usually result from the decreased expression of folylpolyglutamate synthetase (FPGS) or from inactivating mutations in the *FPGS* gene, as well as from the increased expression of folylpolyglutamate hydrolase (FPGH) (7).

Our study focused on a detailed analysis of MTX effects in cell lines derived from two types of pediatric solid tumors, medulloblastoma and osteosarcoma, which were chosen on the basis of their different histogenetic origin and because MTX is typically included in therapeutic protocols for both. The main aim of this study was to analyze the effects of treatment with MTX at concentrations comparable to the MTX plasma levels in patients treated with high-dose or low-dose MTX. Furthermore, an extremely important part of the treatment with high-dose MTX in clinical practice is the administration of LV as an antidote to reduce MTX toxicity in normal cells. Thus, the combined application of MTX and LV was also included in our experiments. An analysis of the expression of genes involved in the mechanisms of resistance to MTX was the final component of our study; the results helped us to elucidate the mechanisms of the various responses to MTX among the examined cell lines.

## Materials and methods

### Cell lines

Two reference cell lines and two cell lines derived in our laboratory were used in this study. Daoy (ATCC HTB-186™) medulloblastoma and Saos-2 (ATCC HTB-85™) osteosarcoma cell lines were purchased from the American Type Culture Collection (Manassas, VA, USA). MBL-02 is an in-house cell line derived previously from a biopsy sample obtained from a 7-year-old girl suffering from desmoplastic medulloblastoma (8). The OSA-08 cell line was newly derived from a biopsy sample obtained from an 11-year-old boy surgically treated for conventional osteosarcoma. The Research Ethics Committee of the School of Medicine (Masaryk University, Brno, Czech Republic) approved the study protocol, and a written statement of informed consent was obtained from each patient or his/her legal guardian.

### Cell culture

Cells were grown in Dulbecco’s modified Eagle’s medium (DMEM) supplemented with 10% (Daoy and Saos-2) or 20% (MBL-02 and OSA-08) fetal bovine serum, 100 IU/ml penicillin, 100 mg/ml streptomycin, and 2 mM glutamine. In addition, the medium for the Daoy cells also contained 1% nonessential amino acids (all cell culture reagents were purchased from PAA, Linz, Austria). Experiments with leucovorin (LV) application were performed in folate-free DMEM (both reagents were purchased from Sigma-Aldrich, St. Louis, MO, USA). Cell culture was performed under standard conditions at 37°C in a humidified atmosphere containing 5% CO_2_.

### Chemicals

MTX (Sigma) was prepared as a stock solution at a concentration of 20 mM in 1 M NaOH (Sigma). This stock solution was diluted in DMEM or folate-free DMEM to obtain the final concentrations used in the experiments. For determination of the IC_50_ value, 7 different concentrations of MTX ranging from 1×10^−4^ to 1×10^2^ *μ*M were tested. For all other experiments, concentrations of 0.1, 1, 10 and 40 *μ*M MTX were used; these concentrations are in the range of MTX plasma levels reached in patients suffering from cancer. The maximum used concentration of MTX, i.e., 40 *μ*M, is comparable with the peak of the MTX plasma concentration achieved during HD-MTX treatment of pediatric solid tumors (4). LV was dissolved in deionized water to prepare a 1 mM stock solution. LV at final concentrations of 10 and 100 nM was prepared in folate-free DMEM.

### MTT assay

To evaluate cell proliferation, an MTT assay to detect the activity of mitochondrial dehydrogenases in living cells was used; 96-well plates were seeded with 1×10^4^ cells/well in 200 *μ*l of culture medium, and the cells were allowed to adhere overnight. The medium was then removed and a new medium containing the selected concentrations of MTX described above or control MTX-free medium was added. The plates were incubated under standard conditions, and LV at the chosen concentrations was added after 42 h. To evaluate changes in cell proliferation, medium with reagents was removed and replaced by 200 *μ*l of DMEM containing 3-[4,5-dimethylthiazol-2-yl]-2,5-diphenyltetrazolium bromide (MTT) at 0.5 mg/ml. The plates were then incubated at 37°C for 2.5 h. Subsequently, the medium was carefully removed, and the formazan crystals were dissolved in 200 *μ*l of DMSO. The absorbance at 570 nm with a reference absorbance at 620 nm was measured using the Sunrise Absorbance Reader (Tecan, Männedorf, Switzerland).

### RT-PCR

Differences in the expression of MTX resistance-related genes in the cell lines under standard conditions were evaluated using RT-PCR. Total RNA was extracted using the GenElute™ Mammalian Total RNA Miniprep kit (Sigma), and its concentration and integrity were determined spectrophotometrically. For all samples, equal amounts of RNA (i.e., 25 ng of RNA per 1 *μ*l of total reaction volume) were reverse transcribed into cDNA using M-MLV (Top-Bio, Prague, Czech Republic) and oligo dT (Qiagen, Hilden, Germany) priming. PCR was carried out in 25 *μ*l reactions containing 12.5 *μ*l of PPP master mix, 0.5 *μ*l of PCR enhancer (both from Top-Bio), 0.5 *μ*M of each primer and 5 *μ*l of diluted cDNA. The primers used for *RFC1*, *DHFR*, *TYMS*, *FPGS*, *FPGH* and *HSP90AB1* are described in Table I. A total of 10 *μ*l of the PCR product was loaded onto a 2% agarose gel stained with Midori Green (Nippon Genetics, Dueren, Germany) and examined after electrophoresis. The optical density was stained and quantified using ImageJ software (9). The data were normalized to *HSP90AB1* expression.

### Flow cytometry

To evaluate changes in the cell cycle, 1.2×10^5^ cells were seeded in 25 cm^2^ Petri dishes and allowed to attach overnight. The cells were then treated with MTX for 3 or 6 days. Both the detached and adherent cells were harvested together, fixed with 70% ethanol and stained with Vindelov’s solution [0.01 M Tris, 10 *μ*g/ml RNase, 50 *μ*g/ml PI and 1 mM NaCl (all from Sigma)] at 37°C for 30 min.

To quantify the rate of apoptosis, 1×10^6^ cells were seeded in 75 cm^2^ Petri dishes and allowed to attach overnight. The cells were treated with MTX for 1 or 3 days. Both the detached and adherent cells were harvested together, fixed with 3% paraformaldehyde (Sigma) at room temperature for 30 min, permeabilized in 0.2% Triton X-100 (Sigma) for 1 min, and incubated with 2% BSA (PAA) for 10 min to block nonspecific antibody binding. The cells were then treated with a rabbit polyclonal anti-cleaved caspase-3 (Asp-175) primary antibody (dilution 1:250, cat. no. 9661; Cell Signaling Technology, Beverly, MA, USA) at 37°C for 60 min. After washing with PBS twice, goat anti-rabbit IgG conjugated with Alexa Fluor^®^ 488 (dilution 1:300, cat. no. A-11008; Life Technologies, Carlsbad, CA, USA) was applied at 37°C for 45 min.

The BD FACSVerse™ flow cytometer with BD FACSuite software (Beckton Dickinson, San Jose, CA, USA) was employed to analyze both the cell cycle and frequency of caspase-3-positive cells at the intervals specified above. Ten thousand events per sample were evaluated in all experiments.

## Results

### Determination of MTX IC_50_

**Figure 1:**
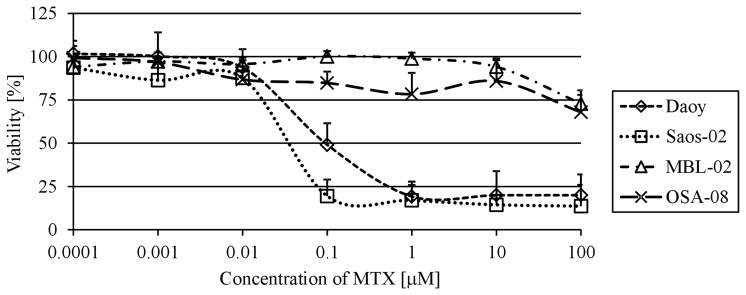
Dose-response curves and IC_50_ values of MTX. Cells were incubated with different doses of MTX for 6 days. Cell viability was analyzed using the MTT assay. Data are presented as the means + standard deviations. x-axis, doses of MTX in *μ*M. y-axis, percentage of viability relative to the untreated cells. MTX, methotrexate.

To confirm that the Daoy and Saos-2 reference cell lines are useful models for the examination of the MTX effects on medulloblastoma and osteosarcoma cells, the IC_50_ values were first determined. Using the MTT assay, we analyzed cell viability at day 6 of MTX treatment in a range of MTX concentrations from 1×10^−4^ to 1×10^2^ *μ*M. Both of these cell lines showed a very similar IC_50_ value: 9.5×10^−2^ *μ*M for Daoy cells and 3.5×10^−2^ *μ*M for Saos-2 cells (Fig. 1). In contrast, neither the MBL-02 medulloblastoma nor the OSA-08 osteosarcoma patient-derived cell lines reached the IC_50_ value within the concentrations of MTX used. The highest concentration of MTX used for experiments with the reference cell lines, i.e., 100 *μ*M, only led to 27 and 32% decreases in viability when compared with the untreated MBL-02 and OSA-08 cells, respectively.

### Effect of MTX and ‛leucovorin rescue’ treatment on cell proliferation

**Figure 2:**
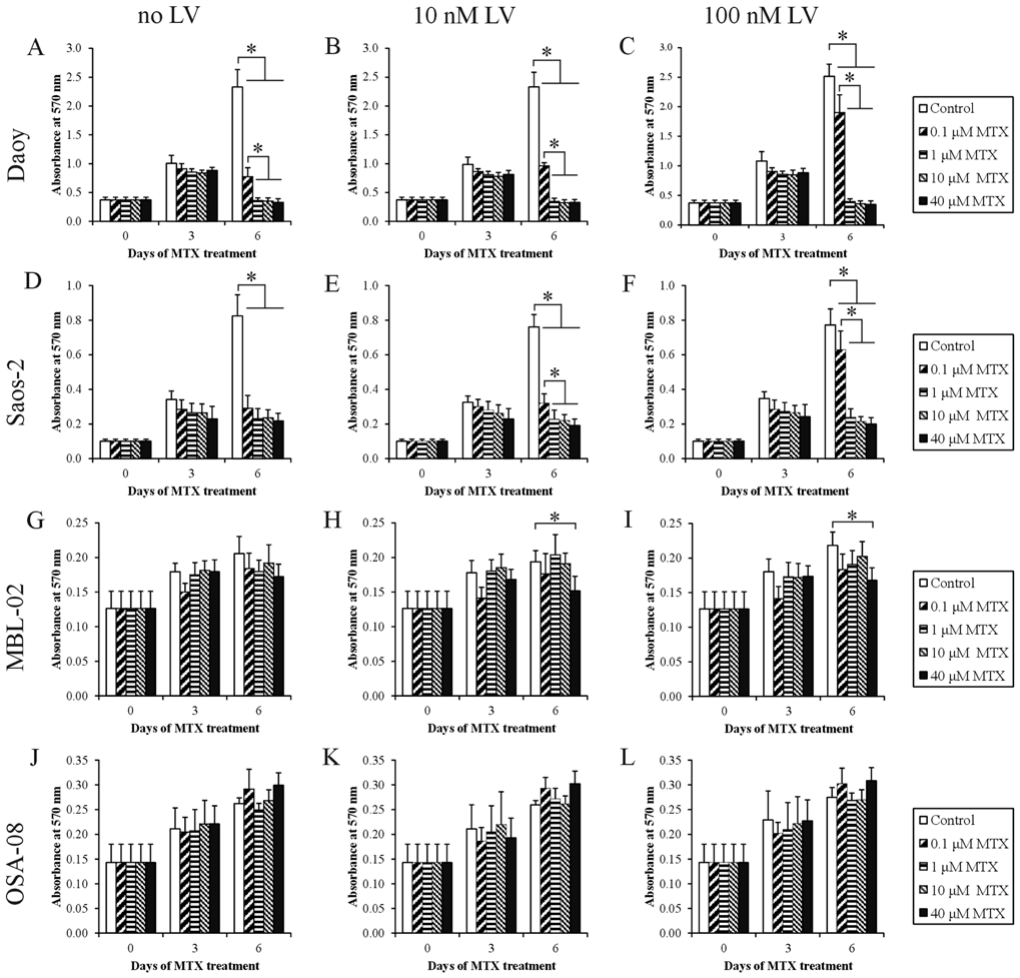
Cell viability after treatment with MTX in combination with LV. Cell viability was measured using the MTT assay before treatment and at day 3 and 6 of incubation with MTX. LV was administered 42 h after the initial treatment with MTX. x-axis, days of treatment. y-axis, absorbance measured at 570 nm. The data represent the means + SEM and were analyzed using ANOVA followed by the Fisher-LSD post-hoc test (^*^p<0.05, significant difference between two treatments). MTX, methotrexate; LV, leucovorin.

To analyze the effects of MTX on cell proliferation, concentrations corresponding to MTX plasma levels were used. Daoy (Fig. 2A) and Saos-2 (Fig. 2D) cell lines showed evident cytostatic effects at day 6 of treatment with MTX at all the chosen concentrations. For Saos-2 cells, no statistically significant differences were observed among all the different MTX treatments. It was also apparent that treatment with 0.1 *μ*M MTX decreased the proliferation of Daoy cells to a significantly lesser extent than the other MTX concentrations. Both the MBL-02 and OSA-08 patient-derived cell lines did not show any marked decrease in the number of viable cells within the concentration interval from 0.1 to 40 *μ*M. Nevertheless, the MBL-02 medulloblastoma cell line (Fig. 2G) appeared to be more sensitive than the OSA-08 osteosarcoma cell line in terms of cell viability (Fig. 2J).

To determine whether the application of LV influences the observed cytostatic effects of MTX, we added LV at two different concentrations, 10 and 100 nM, to the cultivation medium at 42 h after treatment with MTX. The application of 10 nM LV resulted in a slight but statistically significant increase in the proliferation activity of Daoy (Fig. 2B) and Saos-2 (Fig. 2E) cells pretreated with 0.1 *μ*M MTX. In contrast, the use of an elevated concentration of LV, i.e., 100 nM, caused a statistically significant increase in proliferation activity and an inhibition of MTX action in both cell lines pretreated with 0.1 *μ*M MTX (Fig. 2C and F). The cytostatic effects of higher concentrations of MTX were not affected by LV in these cell lines, and the MTX-pretreated in-house cell lines did not respond to the application of LV (Fig. 2H, I, K and L).

### Expression of MTX resistance-related genes

**Figure 3:**
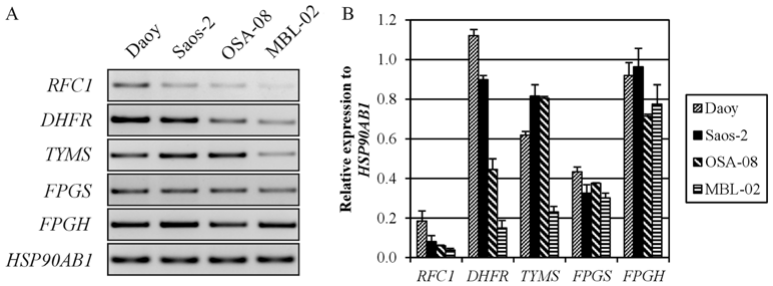
Analysis of gene expression of MTX resistance-related genes. (A) Representative agarose gel. (B) PCR analysis of the mRNA expression of MTX resistance-related genes. The expression of selected genes was quantified using densitometry and was related to the expression of the HSP90AB1 housekeeping gene. x-axis, MTX resistance-related genes. y-axis, expression of the selected genes as related to HSP90AB1. MTX, methotrexate.

To understand the strong differences in MTX toxicity between our in-house cell lines (MBL-02 and OSA-08) and the reference cell lines obtained from ATCC (Daoy and Saos-2), we examined the expression of genes involved in the resistance of tumor cells to MTX (Fig. 3). For this RT-PCR expression analysis, we chose genes encoding the main membrane transporter of MTX, i.e., RFC, two key enzyme targets for MTX, i.e., DHFR and TYMS, and two enzymes catalyzing the glutamylation of MTX, i.e., FPGS and FPGH. The Daoy medulloblastoma cells showed higher relative expression of the *RFC1*, *DHFR* and *TYMS* genes, whereas the expression of these genes was very weak in the MBL-02 medulloblastoma cells. In contrast, both osteosarcoma cell lines displayed similar expression levels of MTX resistance-related genes, with the exception of *DHFR*, the expression level of which was also decreased in the OSA-08 cells.

### Effect of MTX on the cell cycle and cell death

**Figure 4:**
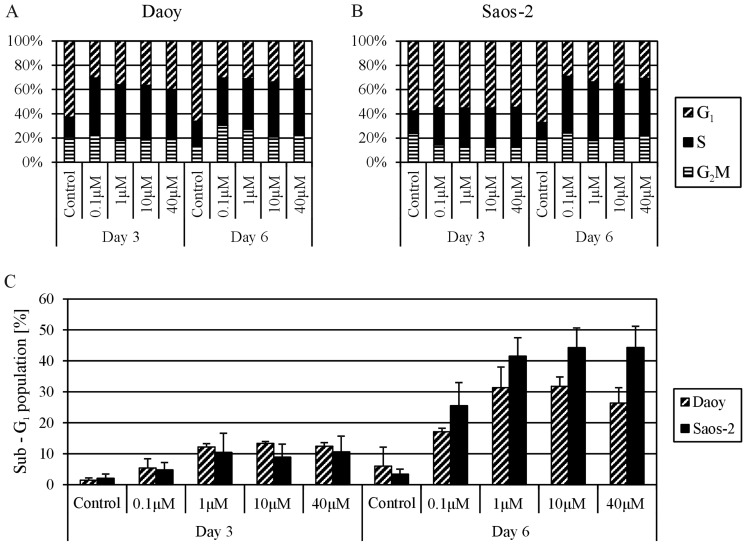
(A and B) Flow cytometric analysis of the cell cycle in Daoy and Saos-2 cells following MTX treatment. Cells were analyzed at day 3 and 6 of incubation with MTX. (C) The percentage of the sub-G_1_ population was evaluated at the same time points. x-axis, doses of MTX and days of treatment (A–C). y-axis, percentage of cells in specific phases of the cell cycle (A and B); percentage of cells in sub-G_1_ phase (C). MTX, methotrexate.

Based on the results of previous experiments, both MTX-responding cell lines, i.e., the Daoy and Saos-2 lines, were chosen for cell cycle analysis. The cells were treated with different concentrations of MTX, and the proportions of cells in sub-G_1_, G_1_, S and G_2_/M phases were determined using flow cytometry at day 3 and 6 of treatment with MTX. All MTX concentrations had the same effect on the distribution of the cell cycle phases; an increase in cells in the S phase was accompanied by a decrease in cells in the G_1_ phase in the treated cell lines compared with the untreated controls. Importantly, this phenomenon was noted sooner in the Daoy cells, at day 3 of MTX treatment (Fig. 4A), but was partially delayed in the Saos-2 cells (Fig. 4B). The analysis of the sub-G_1_ population revealed cytotoxic effects of MTX on both cell lines (Fig. 4C). The population of cells with reduced DNA content markedly increased at day 6 of treatment. Furthermore, this trend was more apparent in the Saos-2 cells; the sub-G_1_ population was >40% in the Saos-2 cells and ~30% in the Daoy cells. The cytotoxic effect of MTX at concentrations ranging from 1 to 40 *μ*M was nearly the same in both cell lines.

**Figure 5:**
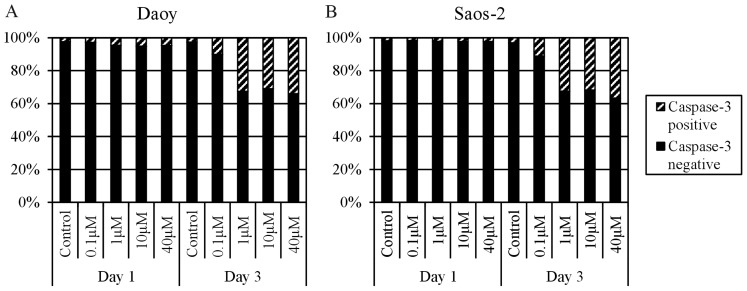
Flow cytometric analysis of caspase-3 positivity in (A) Daoy and (B) Saos-2 cells following MTX treatment. Cells were analyzed at day 1 and 3 of incubation. x-axis, doses of MTX and days of treatment. y-axis, percentage of caspase-3-positive/negative cells in the cell populations. MTX, methotrexate.

To prove whether the increase in the sub-G_1_ proportion after MTX treatment is caused by apoptosis induction, the MTX-treated cell populations were labeled with an anti-active caspase-3 antibody at day 1 and 3 of MTX application. Both cell lines showed a >30% increase in caspase-3-positive cells at day 3 of treatment with 1, 10 or 40 *μ*M MTX. In contrast, treatment with 0.1 *μ*M MTX led to a 7–8% increase in caspase-3-positive cells in comparison to the control cells (Fig. 5).

## Discussion

At present, the standard protocol for cancer treatment with MTX includes the application of HD-MTX, defined as >1 g/m^2^ of body surface, in combination with leucovorin, which enables reaching high plasma concentrations with enhanced anticancer and cytotoxic effects (10).‛Leucovorin rescue’ is administered in a specific time schedule after treatment with MTX, usually from 24 to 42 h, to protect noncancerous proliferating cells from the side effects of MTX. Nevertheless, the toxicity of MTX may induce myelosuppression, mucositis, nephrotoxicity, hepatotoxicity, and, in severe cases, multiorgan failure (11). Although MTX has long been an integral part of many therapeutic regimens, a definite agreement in regards to MTX dosage and timetables and/or LV treatment is still lacking (12).

The main aim of this study was to analyze the effects of MTX on cell lines derived from two types of pediatric solid tumors, medulloblastoma and osteosarcoma, and to determine how these cell lines respond to doses of MTX that correspond to concentrations in a patient’s plasma during administration in clinical practice.

Daoy medulloblastoma and Saos-2 osteosarcoma cell lines were chosen as reference cell lines for this study and were compared to two other cell lines that were derived in our laboratory from these tumors. The Saos-2 osteosarcoma cells were apparently more sensitive to treatment with MTX than the Daoy cells, as revealed by determination of the IC_50_ value (Fig. 1); however, the IC_50_ value obtained for both of these cell lines was within a similar range of concentrations, i.e., 10^−8^ M MTX. These results are in accordance with those obtained by other research groups (5,13). The negative effect of MTX on cell proliferation was clearly evident at day 6 of treatment (Fig. 1) and importantly was in the same concentration range, from 1 to 40 *μ*M, for both of these cell lines. In contrast, only a slight cytotoxic effect of 0.1 *μ*M MTX was able to be reverted by LV (Fig. 2). This finding can be explained by an incomplete inhibition of DHFR by a concentration lower than 1 *μ*M of MTX, as described by Assaraf *et al* (14) on the basis of computational simulation.

Both of our in-house cell lines, i.e., MBL-02 medulloblastoma and OSA-08 osteosarcoma cell lines, appeared to be strongly resistant to MTX; 100 *μ*M MTX did not induce a 50% inhibitory effect in these cells. One of the possible explanations for this difference is the low proliferation rate of these tumor cells in comparison with the reference cell lines (15). No observable effect of LV in these cell lines could be explained by the same mechanisms since treatment with MTX is targeted to quickly proliferating tumor cells.

Other possible specific mechanisms of resistance include impaired transmembrane uptake, alterations in the expression or activity of target enzymes, or impaired intracellular polyglutamylation as a determining process of drug efficacy (6). The *RFC1* gene, which encodes the transmembrane solute carrier and is considered to be a main MTX transporting pathway to the cytoplasm (16), was only expressed weakly in our in-house cell lines (Fig. 3). Consequently, the low levels of RCF may have caused a decrease in MTX intracellular availability. Conversely, high levels of *DHFR* expression were detected in both the Daoy and Saos-2 cells compared with these levels in the in-house cell lines (Fig. 3). On the one hand, increased levels of DHFR have been commonly observed in cells exhibiting an MTX-resistant phenotype (17). On the other hand, this key enzyme involved in the *de novo* synthesis of purine and pyrimidine precursors plays a critical role in cell growth and proliferation, and its high expression in rapidly proliferating cells is thus expected (2,18). Although the expression levels of *TYMS* in both osteosarcoma cell lines were identical, marked differences in the expression of this gene were detected between the medulloblastoma cell lines, with higher levels of *TYMS* found in Daoy cells with higher proliferation activity (Fig. 3). The product of the *TYMS* gene catalyzes dUMP conversion into dTMP and thus provides the sole source of deoxythymidylate for DNA biosynthesis (6). In fact, ectopic TYMS expression has been shown to promote cell proliferation *in vitro*, and the high expression of TYMS in tumor tissue is also associated with poor clinical outcome in some types of cancers (19). Another mechanism of resistance to MTX affects the ratio of FPGS/FPGH since polyglutamylated MTX has a substantially longer half-life than monoglutamated MTX (20). Nevertheless, all four cell lines showed similar expression levels of both *FPGS* and *FPGH* (Fig. 3); thus, the differences in MTX effects on cell proliferation were not caused by changes in MTX polyglutamylation.

The flow cytometric analysis of the MTX-sensitive cell lines, i.e., Daoy and Saos-2 cells, clearly confirmed the two main effects of MTX on tumor cells that are responsible for its ability to restrict cell proliferation. First, the cell cycle was arrested in S-phase due to the depletion of nucleotide precursors; our results showed apparent MTX-induced cell cycle arrest in S-phase (Fig. 4). Similar findings were previously described for cell lines derived from adrenocortical carcinoma (21), glioblastoma (22) and lung carcinoma (23). Notably, we did not observe any significant differences in the effects of the MTX concentrations ranging from 0.1 to 40 *μ*M on the distribution of cell cycle phases (Fig. 4). Secondly, the induction of cell death detected as the sub-G_1_ fraction following treatment with MTX was also involved in proliferation failure (Fig. 4). Furthermore, we noted a marked difference between treatment with 0.1 *μ*M MTX and the treatments with other concentrations (Fig. 4), and these results were in accordance with those achieved by the detection of activated caspase-3-positive cells (Fig. 5). Caspase-3-dependent/p53-independent apoptosis induced by MTX was previously described in MCF-7 breast carcinoma cells (24). The apoptosis induced in the Saos-2 and Daoy cells was also p53-independent since Saos-2 cells do not express p53 (25) and the C242F mutation in the *TP53* gene, which disables the transactivation function of the p53 protein, was proven in Daoy cells (26,27),

To summarize, our results showed that treatment with MTX significantly decreased proliferation activity, inhibited the cell cycle at S-phase and induced apoptosis in the Daoy and Saos-2 reference cell lines. Such effects apparently belong to the DHFR-mediated mechanism of MTX action and are based on the depletion of purine and pyrimidine precursors necessary for the biosynthesis of nucleic acids. Importantly, we noted no difference in these effects after treatment with various doses of MTX ranging from 1 to 40 *μ*M. These findings suggest the possibility of achieving the same outcome with the application of low-dose MTX, which is an extremely important result, particularly for clinical practice, and may explain the lack of clinical advantage of HD-MTX in children with advanced lymphoblastic lymphomas vs. low doses (28). Moreover, the combined application of MTX together with LV did not produce any detectable effect, with exception of a partial reduction in MTX toxicity after the use of the lowest concentration of MTX, i.e., 0.1 *μ*M.

## Figures and Tables

**Table I:** Sequences of the primers used for RT-PCR.

Gene	Primer sequences	Product (bp)
RFC1	F: 5′-GCGGGCTTCGTGAAGATC-3′	330
	R: 5′-CTGGAACTGCTTGCGGAC-3′	
DHFR	F: 5′-CAGAACATGGGCATCGGCAAGAACG-3′	328
	R: 5′-AAACAGAACTGCCACCAACTATCCA-3′	
TYMS	F: 5′-CGGGAGACATGGGCCTCGGT-3′	353
	R: 5′-GCATCCAGCCCAACCCCTAA-3′	
FPGS	F: 5′-CACTGGGACGAAGGGGAA-3′	322
	R: 5′-GTCATAAGCCCCGCCAAT-3′	
FPGH	F: 5′-AAAGTACTTGGAGTCTGCAGGTGC-3′	327
	R: 5′-TGCAATTGACCTCCAGTGAAGTTCA-3′	
HSP90AB1	F: 5′-CGCATGAAGGAGACACAGAA-3′	169
	R: 5′-TCCCATCAAATTCCTTGAGC-3′	

## Abbreviations

DHFR: dihydrofolate reductase
FPGH: folylpolyglutamate hydrolase
FPGS: folylpolyglutamate synthetase
LV: leucovorin
MTX: methotrexate
RCF: reduced folate carrier
THF: tetrahydrofolate
TYMS: thymidylate synthase
